# Antifungal Susceptibility Profile of *Candida* Species Isolated From Water Treatment Unit and Urine From Patients Undergoing Hemodialysis in General Hospital and University Teaching Hospital in Yaounde, Cameroon

**DOI:** 10.1155/cjid/5583130

**Published:** 2025-09-09

**Authors:** Tidding Ashley Ambock, Bitoungui Valentina Josiane Ngo, Guy Sedar Singor Njateng, Kamga Hortense Gonsu, Mbetyoumoun Heroine Mfouapon, Folefack Francois Jerome Kaze, Tomta Aristide Eric Nono, Kueti Flora Mafonang

**Affiliations:** ^1^Department of Microbiology, Hematology and Immunology, Faculty of Medicine and Pharmaceutical Sciences, University of Dschang, P.O. Box 96, Dschang, Cameroon; ^2^National Laboratory of Public Health, Ministry of Public Health, Yaounde, Cameroon; ^3^Department of Biochemistry, Faculty of Science, University of Dschang, P.O. Box 67, Dschang, Cameroon; ^4^Department of Pharmaceutical Sciences, Faculty of Medicine and Pharmaceutical Sciences, University of Dschang, P.O. Box 96, Dschang, Cameroon; ^5^Faculty of Medicine and Biomedical Sciences of University of Yaounde I, P.O. Box 1364, Yaounde, Cameroon; ^6^University Teaching Hospital, P.O. Box 1364, Yaounde, Cameroon; ^7^General Hospital of Yaounde, P.O. Box 5408, Yaounde, Cameroon

**Keywords:** antifungal susceptibility profile, candiduria, hemodialysis, water contamination, Yaounde, Cameroon

## Abstract

**Background:** Improper maintenance of water used for hemodialysis poses a serious risk for patients by allowing the growth of biological contaminants like *Candida* species in the water system. These contaminants can be transmitted to patients through hemodialysis, exposing them to an increased risk of candidiasis. The objective of this study was to determine the antifungal susceptibility profile of *Candida* species isolated from water treatment units and urine from patients undergoing hemodialysis at GHY and UTHY in Yaounde, Cameroon.

**Methods:** This cross-sectional descriptive study was conducted from February 2024 to May 2024. Urine samples were collected from 132 consenting hemodialysis patients using a questionnaire, cultured on Sabouraud plus chloramphenicol agar. Water samples were collected every two days from different sites in the hemodialysis circuits; input, pretreatment, reverse osmosis (RO), RO output of both hospitals, filtered through membranes deposited on SC, and incubated at 35°C–37°C. Positive samples were cultured on CHROMagarTM to identify *Candida* species, which were tested against antifungals. SPSS V29.0.1.0 and Excel 2019 were used for statistics.

**Results:** Of the patients, 17 (12.88%) had candiduria with *C. auris* (35.30%) most prevalent. Of 180 water samples, *C. tropicalis* (44.4%) was isolated from input water, while *C. glabrata* (22.2%) and *C. parapsilosis* (22.2%) were found in input and pretreated water. *C. albicans* (11.1%) was isolated at the RO output. Antifungal susceptibility testing of 35 *Candida* isolates showed that water isolates of *C. glabrata*, *C. parapsilosis*, and *C. albicans* were 100% susceptible to fluconazole and ketoconazole. *C. tropicalis* from water exhibited resistance to fluconazole. *C. auris* from urine was resistant to all antifungals tested. None were susceptible to itraconazole or amphotericin B.

**Conclusion:** This study emphasizes the critical need for rigorous maintenance and monitoring of water used in hemodialysis to prevent transmission of antifungal-resistant *Candida* to vulnerable patients.

## 1. Introduction

Hemodialysis is a specific form of dialysis which uses a semipermeable membrane to filter blood and remove waste products, excess water, and electrolytes [1]. Hemodialysis is the predominant form of renal replacement therapy (RRT), with approximately 4 million individuals worldwide relying on this life-saving treatment [2]. The global demand for RRT is expected to rise significantly in the coming years, with the number of individuals requiring such therapy projected to increase from 3.3 million in the current year to 5.4 million by 2030, with the most substantial increase occurring in developing countries [3]. This trend is clearly illustrated in the Cameroon's city capital, where 700 patients were recorded in the two main hospitals as undergoing hemodialysis [4].

One of the major concerns for patients undergoing hemodialysis is their compromised immune system, which makes them highly prone to different types of infections, including fungal infections [5]. Fungal infections can be a significant cause of morbidity and mortality in this patient population [6]. It has been reported that 3.7 million deaths worldwide are due to fungal infections [7]. The water used for hemodialysis is derived from the public water supply, a potential source of pathogens as patients are exposed to approximately 400 L of water per week through the dialyzer's semipermeable membrane [8, 9]. This is particularly concerning since water and soil are natural habitats for fungi and patients undergoing hemodialysis with their weakened immune system are susceptible to opportunistic microorganisms, especially *Candida* species, which are the fourth most common cause of hospital-acquired infections [10].

Studies have indeed shown a higher incidence of fungal infections, particularly *Candida-*related infections, among hemodialysis patients compared with the general population. Research has found a 15% prevalence of *Candida* urinary tract infections in Iranian patients with renal failure undergoing hemodialysis [11] and a 47.2% prevalence of *Candida* species isolated from water reservoirs used in hemodialysis units in Brazil [12]. The rise of antifungal resistance has emerged as a critical public health issue and is now an emerging concern worldwide with *Candida* species demonstrating the ability to develop resistance to multiple drugs [13–15]. This has limited treatment options, leading to a shift toward antifungal-resistant species [16].

As the need for hemodialysis grow, particularly in developing countries and given the significant burden of fungal infections in this patient population, and the potential role of water quality, there is a need for extensive research on the antifungal susceptibility profile of *Candida* species in the water reservoirs and patient isolates, especially in Africa and Cameroon. These insights will inform targeted interventions and treatment strategies to address this critical healthcare challenge.

## 2. Material and Methods

### 2.1. Study Design and Settings

A cross-sectional descriptive study was conducted over a period of four months from February 2024 to May 2024 in two hospitals in Yaounde: General Hospital Yaoundé (GHY) and University Teaching Hospital Yaoundé (UTHY). The study received ethical clearance from the Regional Ethics Committee and administrative approval from GHY and UTHY. The hospitals are key facilities in Cameroon providing healthcare to patients undergoing hemodialysis with approximately 500 patients.

### 2.2. Selection Criteria of Participants

All patients undergoing hemodialysis followed at general hospital and university teaching hospital were eligible for the study. All water systems that were part of the hemodialysis water treatment units used at GHY and UTHY were included in the study.

The inclusion criteria for patients comprised individuals who had undergone hemodialysis for at least 2 months and those who were nonanuric while receiving hemodialysis. For water, the inclusion criteria specified that the water treatment units must be part of the dialysis water treatment system, including storage tanks and distribution pipelines, and must currently be in use for active hemodialysis treatment. Exclusion criteria were patients receiving antifungal treatment, those who withdrew consent during the study, and water samples that were broken or had incomplete labeling.

### 2.3. Minimum Sample Size

The minimum required sample size was calculated using the Lorentz (Cochran) formula for estimating prevalence based on three parameters: a 95% confidence level (*Z* = 1.96), an expected prevalence (*p*), and a margin of error (*d*).

For the patients undergoing hemodialysis, we used an expected candiduria prevalence of a 2.7% according to the prevalence of candiduria in patients undergoing hemodialysis obtained in Addis Ababa, Ethiopia [17], which yielded a minimum sample size of 41 patients.

For the water samples, we assumed a *Candida* prevalence of 3.2% based on a study done in Brazil [18], applying the same confidence level and margin of error, which led to a minimum sample size of 48 water samples.

### 2.4. Sampling Method

The study used an exhaustive, census-style sampling method, collecting urine samples from all eligible hemodialysis patients at GHY and UTHY. Informed consent and questionnaires (supporting information: Questionnaire (English and French version) (available here)) were used to ensure comprehensive representation of the patient population. Additionally, water samples were collected from Camwater, the municipal supply, to assess microbial characteristics before treatment. This pretreatment analysis provided insights into the filtration and disinfection processes affecting *Candida* species levels. Finally, analyzing water from the reverse osmosis (RO) system evaluated the purification effectiveness and the quality of the pipes transporting the treated water to dialysis machines.

### 2.5. Sample Collection

#### 2.5.1. Urine Sample

Once patients agreed to participate, they were asked to provide a midstream urine sample of at least 10 mL in a sterile container. They received detailed instructions, including handwashing and cleaning the genital area with Dakin's solution. The collection involved discarding the first portion of urine, capturing the midstream in the sterile container, and discarding the final part. The container, a 50 mL leak-proof plastic cup, was securely closed and returned with a label containing the patient's name, sex, age, and a unique identification code matching the questionnaire.

#### 2.5.2. Water Sample

Aseptic collection of 500-mL water samples was conducted by the principal investigator during active hemodialysis. Water was allowed to flow for 2 min before samples were collected in sterile containers from various points: 500 mL each from municipal water supply at the entry point, pretreated water, and RO at GHY, as well as municipal water supply, RO, and outlet of RO at UTHY. Each container was labeled with the sample type, hospital initials, date, and time of collection.

### 2.6. Mycological Analysis

#### 2.6.1. Culture

Urine and water samples were cultured on Sabouraud dextrose agar supplemented with chloramphenicol to support yeast growth while inhibiting bacteria. Urine samples from patients undergoing hemodialysis were inoculated onto labeled Petri dishes and incubated at 35°C–37°C for 18–24 h, with visible *Candida* colonies indicating presence. Quality control involved pH verification, temperature checks, and fertility testing with *Candida albicans* strain L26, NR-29445 as a positive control. Water samples were filtered through a 0.45-μm membrane. The nitrocellulose membrane was later on placed on Sabouraud dextrose agar and incubated for two days at 35°C–37°C. Colonies were reported as mean colony forming units (CFU/mL), with sterile water for injection was used as control.

#### 2.6.2. Identification

After culturing on Sabouraud agar with chloramphenicol, *Candida* species appeared as creamy, smooth, white colonies with a slightly raised shape. These colonies were then observed under a microscope at 10x and 40x magnification, showing oval, off-white yeasts with a budding chain appearance. Confirmed colonies were cultured on CHROMagar™ *Candida*, which enabled species-specific coloration through the hydrolysis of a chromogenic substrate by a hexosaminidase enzyme. Quality control involved testing pH, temperature, and fertility using *Candida albicans* strain L26 as a reference. The color characteristics on CHROMagar™ included *Candida albicans* (dark green to bluish-green), *Candida tropicalis* (dark blue), *Candida glabrata* (dark pink), *Candida krusei* (pink with rough edges), *Candida parapsilosis* (light pale), and *Candida auris* (very light green).

#### 2.6.3. Antifungal Susceptibility Testing

Antifungal susceptibility testing was conducted using disk diffusion according to CLSI M-44 guidelines. After 24 h of culturing *Candida* species on Sabouraud agar with chloramphenicol, colonies around 5 mm in diameter were suspended in 5 mL of sterile 0.145 mol/L saline, achieving a 0.5 McFarland standard for standardized inoculum density. A sterile swab was dipped into the suspension and used to evenly streak Mueller–Hinton agar supplemented with 2% glucose, rotating every 30° for complete coverage. Antifungal disks (amphotericin B, nystatin, ketoconazole, fluconazole, itraconazole, and clotrimazole) were placed on the agar and incubated at 35°C–37°C for 18–24 h. After incubation, the zones of inhibition around each disk were measured to assess antifungal efficacy.

## 3. Results

A total of 136 patients undergoing hemodialysis participated in the study, with 80 (60.6%) from GHY and 52 (39.4%) from UTHY. Of these, 132 (97.1%) met the inclusion criteria, while 4 (2.9%) were excluded due to incomplete requirements. A total of 180 water samples were collected from both hospitals, with 90 samples from each, including sources like Camwater and RO. Participants' ages ranged from 20 to 80 years, with a mean age of 48.71 ± 14.50 years, 70.5% were married, and 67.4% held higher education degrees. Comorbidities were common, with 65.2% having hypertension, 41.7% diabetes, and 7.1% living with HIV. The duration of dialysis varied, with 42.4% undergoing treatment for less than 1 year. Most participants (68.9%) reported drinking mineral water, while others consumed Camwater or forage water.

**Figure 1:**
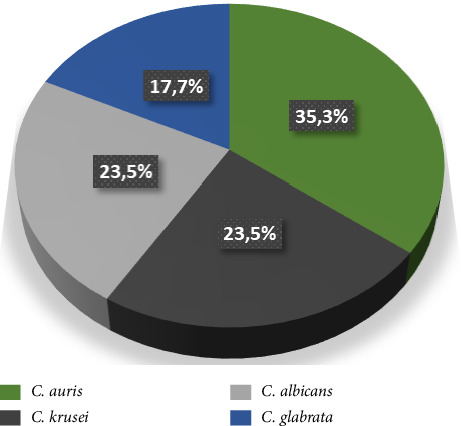
Distribution of *Candida* species isolated from urine of patients undergoing hemodialysis.

The study involved 132 hemodialysis patients, with 17 (12.88%) diagnosed with candiduria. The most prevalent *Candida* species was *C. auris* at 35.3% (*n* = 6), followed by *C. albicans* (23.5%), *C. glabrata* (17.7%), and *C. krusei* (23.5%) (Figure 1). Factors associated with candiduria were assessed using both univariate and multivariate odds ratios, revealing that age (20–39 years) and sex were significant in the univariate model but not in the multivariate model after adjustment.

A total of 180 water samples were collected from the hemodialysis units: 90 from GHY and 90 from UTHY (Table 1). *Candida* was detected in 16 (8.9%) of these samples, with a higher positivity rate in GHY. Specifically, 8 out of 60 Camwater samples from GHY tested positive (13.3%), while 2 out of 30 pretreatment samples were positive. In contrast, at UTHY, 10 out of 60 Camwater samples (16.7%) also tested positive, with 4 out of 30 RO outlet samples showing *Candida* presence. Overall, mean CFU/L were calculated to assess the microbial load. In GHY, the water samples yielded a higher prevalence of *C. tropicalis* (66.7%) and *C. glabrata* (16.7%), while UTHY also had *Candida* species detected but with different distribution patterns. Notably, no *Candida* species were found in the RO permeate from either hospital, although two isolates of *C. albicans* were detected at the RO outlet in UTHY (Table 2).

The antifungal susceptibility profile of *Candida* species isolated from water reservoirs and hemodialysis patients was evaluated using six antifungal agents against 35 *Candida* isolates (Table 3). The isolates displayed high susceptibility rates to most azoles, except for itraconazole, to which none of the isolates were susceptible (0.0%). Both *C. parapsilosis* and *C. albicans* from water samples, as well as *C. glabrata* from urine, showed 100% susceptibility to fluconazole and ketoconazole. Clotrimazole also exhibited good susceptibility rates against *C. parapsilosis* and *C. albicans* from water reservoirs. Conversely, nystatin demonstrated a low overall susceptibility rate of 22.9%. Amphotericin B showed high resistance, with no isolates exhibiting susceptibility (0.0%). Additionally, some antifungals, including itraconazole, amphotericin B, clotrimazole, and nystatin, had intermediate or dose-dependent susceptibility in over 20% of the isolates (34.3% for itraconazole, amphotericin B, and clotrimazole; 28.5% for nystatin).

## 4. Discussion

This study was the first study conducted in Cameroon. It aimed at to describe the antifungal susceptibility profile of *Candida* species isolated in water treatment units and urine of patients undergoing hemodialysis. It aimed to determine the prevalence of candiduria among patients undergoing hemodialysis, distribution of *Candida* species in water treatment units used for hemodialysis, and the antifungal susceptibility profile. The prevalence of candiduria obtained was 12.88%, which was similar to that obtained in Iran who determined the prevalence of candiduria in 253 patients with renal failure undergoing hemodialysis in Imam Reza hospital and obtained a result of 15.8% [19]. The prevalence of candiduria obtained from this study was higher than 2.7% prevalence of candiduria among 222 patients undergoing hemodialysis in Addis Ababa, Ethiopia [17]. This difference may be due to the fact that this study population had 72.7% of the patients who were more than 40 years old, whereas in the study done in Ethiopia, less patients were older than 40 years. A total of 180 water samples were collected from two hospitals at various points, as outlined in the methodology. Of the 180 samples, 16 samples were found to contain *Candida* species. Of the 60 water samples from Camwater, 12 isolates of different *Candida* species were found: *C. tropicalis* (66.6%), *C. glabrata* (16.7%), and *C. parapsilosis* (16.7%). Of the 30 water samples collected from the pretreatment, four isolates of two different species were found, *C. glabrata* (50.0%) and *C. parapsilosis* (50.0%). No *Candida* species was found in the permeate (RO), but two isolates of *C. albicans* were detected at the outlet of the RO. The results obtained from this study were higher than that obtained in Brazil, who evaluated the occurrence of fungi in dialysis water and dialysate and their biofilm production capacity and found just one *Candida* species which was left unidentified [18]. The results obtained were similar to another study done in Brazil who studied on yeast isolation and identification in water used in a Brazilian hemodialysis unit [12]. They collected 30 samples from the water supply network and found 10 isolates of *Candida* species, which were *C. parapsilopsis* (80.0%) and *C. guilliermondii* (20.0%) [12]. The reason *C. tropicalis* was found in Camwater but not found in pretreated water unlike *C. glabrata* and *C. parapsilopsis* which were found in Camwater and pretreated water may be due to the difference in the extracellular vesicles (EVs) produced. *C. tropicalis* produces EVs averaging nearly 245 nm in diameter whereas EVs from *C. glabrata* and *C. parapsilosis* generally fall below 180 nm in size. Additionally, EVs derived from *C. tropicalis* are notably richer in proteins, lipids, and carbohydrates compared with those from the other two species [17]. Because of their larger size and dense macromolecular content, *C. tropicalis* EVs may be more prone to physical disruption or removal during pretreatment processes in water treatment systems (e.g., filtration and sedimentation). Furthermore, maintaining such EVs requires a minimal availability of ions, as extensive chitin synthesis and remodeling in *C. tropicalis* cell walls significantly increases ionic demand, which pretreated condition (ion depleted) water cannot sustain [20]. As a result, *C. tropicalis* may struggle to persist in those environments, whereas species producing smaller, simpler EVs may survive more readily. This may be the reason why even though we had the largest percentage of *C. tropicalis* in the municipal water supply, none was identified in pretreated water contrarily to *C. parasilopsis* and *C. glabrata.*

The variation in identified species and isolate numbers compared with other studies may arise from differences in geographical location, sampling methods, water sources, treatment processes, and the effectiveness of disinfection procedures. Additionally, factors like water temperature, pH, and residual disinfectants can influence the growth of specific *Candida* species in water reservoirs, making direct comparisons challenging [21]. These findings showed that no *Candida* species were found in the permeate, which is the final treated water used for dialysis unlike the results in Brazil which showed the presence of 11 *Candida* isolates in the permeate [12]. However, *Candida albicans* was discovered in the outlet of the RO system in the University Teaching Hospital, which is the permeate that flows through the polyvinyl chloride (PVC) pipes connected to the dialysis machines. *Candida* species found in the outlet of RO even though absent in the permeate can be due to the presence of organotin compounds, which are materials used to improve the thermal and ultraviolet (UV) stability of the pipes [22, 23]. Some *Candida* species possesses triorganotin hydrolase that degrades organotin and used the product for their growth, forming biofilms that make them adhere to the PVC pipes [23]. A total of 35 *Candida* isolates representing various species were tested for antifungal susceptibility. The results indicated an overall susceptibility of 74.0% to ketoconazole and 60.0% to fluconazole. These findings align with previous studies on *Candida* isolates from clinical specimens in Yaounde and Garoua, Cameroon, which reported similar susceptibility rates among patients living with HIV and pregnant women with vulvovaginal candidiasis [24]. *C. parapsilosis* isolated from water reservoirs exhibited a high susceptibility rate, with 100.0% susceptibility to clotrimazole, fluconazole, and ketoconazole. This contrasts with findings in Bafoussam, Cameroon, who reported low susceptibility rates (< 20.0%) to azoles in vulvovaginal candidiasis [25]. *C. tropicalis* from water reservoirs demonstrated the highest resistance to fluconazole, attributed to large EVs formed during biofilm development, which reduce susceptibility [20]. *C. auris* isolated from urine was highly resistant to nearly all antifungals, potentially due to the overexpression of efflux pumps from the *Candida* drug resistance (CDR) and multidrug resistance (MDR) gene families, which transport antifungal drugs out of the cell [26]. However, this species showed high susceptibility (100.0%) to clotrimazole, ketoconazole, and fluconazole, suggesting these could be effective treatment options for polyene-resistant infections. Resistance to amphotericin B was observed in over 75.0% of the isolates, including *C. auris*, *C. krusei*, *C. parapsilosis*, and *C. albicans*. This trend aligns with findings regarding antifungal resistance in Cameroon [24, 26]. The resistance is likely due to decreased binding of amphotericin B to ergosterol in plasma membranes. *C. glabrata* isolated from water reservoirs showed concerning resistance (75.0%) to itraconazole and amphotericin B, while *C. parapsilosis* exhibited high susceptibility to azoles. Notably, the study found 100.0% resistance of all isolates to amphotericin B, which contrasts with earlier studies in China, Saudi Arabia, and Iran, where various susceptibility rates were reported [27, 28]. Overall, these results highlight the increasing prevalence and antifungal resistance of nonalbicans *Candida* species globally.

## 5. Conclusion

This study evaluated the antifungal susceptibility of *Candida* species from water reservoirs and urine of hemodialysis patients at a hospital in Yaoundé. Among 132 patients, candiduria prevalence was 12.88%, with *C. auris* as the most isolated species (35.3%). From 180 water samples, 16 tested positive for *Candida*, primarily *C. tropicalis* (66.6%), *C. glabrata*, and *C. parapsilosis*. All isolates showed 100% susceptibility to fluconazole and ketoconazole, except *C. tropicalis*, which had lower susceptibility. Itraconazole and amphotericin B exhibited 0% susceptibility, while clotrimazole was effective against *C. parapsilosis* and *C. albicans*. Overall, the findings highlight the high prevalence of *C. auris* and concerning low susceptibility patterns, necessitating further research and infection control measures.

## Figures and Tables

**Table 1:** Variation of the presence of *Candida* species in water reservoirs according to hospitals and site of collection.

Variables	Modalities	Overall *n* (%)	Positivity to *Candida n* (%)	*p*-value2
Overall	180	16 (8.9)		

Hospital	UTHY	90 (50.0)	8 (8.9)	1.000
	HGY	90 (50.0)	8 (8.9)	

Site of sampling	Camwater	60 (33.3)	10 (16.7)	0.010
	Pretreatment	30 (33.3)	4 (13.3)	
	Reverse osmosis	60 (66.6)	0 (0.0)	
	Outlet of RO	30 (33.3)	2 (6.7)	

*Note: n* = number of water samples/number of isolates.

**Table 2:** Distribution of *Candida* species in water reservoirs collected from hemodialysis unit according to sites of collection.

Collection sites	*C. parapsilopsis*		*C. glabrata*		*C. tropicalis*		*C. albicans*	
	*n*	%	*n*	%	*n*	%	*n*	%
Camwater	2	16.7	2	16.7	8	66.7	0	0
Pretreatment	2	50.0	2	50.0	0	0	0	0
Permeate (RO)	0	0	0	0	0	0	0	0
Outlet of RO	0	0	0	0	0	0	2	100.0

*Note: n* = number of isolates.

**Table 3:** Antifungal susceptibility profile of *Candida* species isolated from water treatment units and urine.

Antifungals	Cau	Ck	Ct	Cp	Cg		Ca		Total (%)
	U (*n* = 6) %	U (*n* = 4) %	W (*n* = 8) %	W (*n* = 4) %	U (*n* = 3) %	W (*n* = 4) %	U (*n* = 4) %	W (*n* = 2)	
Amphotericin B									
S	0.0	0.0	0.0	0.0	0.0	0.0	0.0	0.0	0.0
I	0.0	16.7	75.0	25.0	66.7	25.0	25.0	0.0	34.3
R	100.0	83.3	25.0	75.0	33.3	75.0	75.0	100.0	65.7
Itraconazole									
S	0.0	0.0	0.0	0.0	0.0	0.0	0.0	0.0	0.0
I	50.0	50.0	12.5	0.0	66.7	25.0	50.0	50.0	34.3
R	50.0	50.0	87.5	100.0	33.7	75.0	50.0	50.0	65.7
Nystatin									
S	0.0	33.3	62.5	0.0	33.3	0.0	0.0	0.0	22.9
I	75.0	50.0	0.0	50.0	33.3	75.0	75.0	0.0	48.6
R	25.0	16.7	37.5	50.0	33.3	25.0	25.0	100.0	28.5
Clotrimazole									
S	50.0	33.3	37.5	100.0	0.0	25.0	50.0	100.0	45.7
I	50.0	66.7	12.5	0.0	100.0	25.0	25.0	0.0	34.3
R	0.0	0.0	50.0	0.0	0.0	50.0	25.0	0.0	20.0
Fluconazole									
S	25.0	66.7	50.0	100.0	0.0	100.0	50.0	100.0	60.0
I	0.0	0.0	0.0	0.0	66.7	0.0	0.0	0.0	5.7
R	75.0	33.3	50.0	0.0	33.3	0.0	50.0	0.0	34.3
Ketoconazole									
S	0.0	83.3	75.0	100.0	100.0	100.0	50.0	100.0	74.3
I	75.0	16.7	12.5	0.0	0.0	0.0	50.0	0.0	20.0
R	25.0	0.0	12.5	0.0	0.0	0.0	0.0	0.0	5.7

*Note:* Cau = *C. auris*, Ct = *C. tropicalis*, Ck = *C. Krusei*, Cp = *C. parapsilosis*, Cg = *C. glabrata*, Ca = *C. albicans*, U = urine, W = water, *n* = number of isolates.

## Data Availability

All data will be available on request from the corresponding author.
