# Supplementary material for: Antifungal Susceptibility Profile of Candida Species Isolated From Water Treatment Unit and Urine From Patients Undergoing Hemodialysis in General Hospital and University Teaching Hospital in Yaounde, Cameroon
Source: Can J Infect Dis Med Microbiol. 2025 Sep 9;2025:5583130. doi: 10.1155/cjid/5583130 (PMC12440637; doi:10.1155/cjid/5583130)
Supplement: Supporting Information — Additional supporting information can be found online in the Supporting Information section. [file 5583130.f1.docx]

**QUESTIONNAIRE**

1. **Sociodemographic data**

| **Name of Hospital** | | **Code** |
| --- | --- | --- |
| **Age** | **Sex:** Female________ Male___________ | |
| **Marital Status:** Single____ Married___ Divorced___ Widowed**___** Partnership___ | | |
| **Level of Education:** None_____ Primary_____ Secondary____ Higher_____ | | |
| **Profession:** | | |
| **Zone of Residence:** Town______________________ Quarter______________________ | | |

1. **Medical History**

**Answer the following questions by Ticking the answer that is most appropriate for you where necessary. In Question 4, you can tick more than one answergoo**

| 1. **How long have you been on hemodialysis treatment**? |
| --- |
| 1. **Which disease are you suffering from**?   End Stage Kidney Disease Diabetic Nephropathy Glomerulonephritis Polycystic Kidney Disease Congenital Kidney Disorder Acute kidney failure  If other precise_____________________________________ |
| 1. **How often do you undergo hemodialysis**?   1/ month 2/ month 1/week 2/week 3/week 4/Week |
| 1. **Do you have any of this disease**?   Diabetes Hypertension AIDS |
| 1. **If other Precise_________________________________________** |
| 1. **Have you been diagnosed with Fungi infections this past two weeks**? Yes No |
| 1. **Have you taken any antifungal this past week?** Yes No |
| 1. If yes which?____________________________________________ |
| 1. **How often do you take the antifungals**? Every day once a week once a month |
| 1. **Which type of water do you drink at home**?   Bottled water Forage water Stream water Well water Tap water |
| 1. **How often do you drink water**? <1L/day Atleast 1.5L/day >1.5L/day |
| 1. **Have you recent changes in your hemodialysis catheter?** Yes No |
| 1. **Have you noticed any leaks with your catheter**? Yes No |
| 1. **Have you travelled this month?** Yes No   If Yes, Where To? _________________________________________________ |

1. **Signs and Symptoms**

**Answer the following questions on the signs and symptoms you might have felt by ticking either yes or no**

| **Questions** | **Yes** | **No** |
| --- | --- | --- |
| 1. Have you experienced any lower abdominal pain or discomfort? |  |  |
| 1. Have you had any persistent fever this past 4 weeks? |  |  |
| 1. Have you experienced any itching, redness or irritation in your genital area? |  |  |
| 1. Have you experienced burning sensation while urinating? |  |  |
| 1. Have you noticed unusual cloudiness in your urine? |  |  |
| 1. Have you been feeling tired this last 2weeks? |  |  |

| **Nom de l'hôpital** | | **Code** |
| --- | --- | --- |
| **Age** | **Sexe :** Féminin________ Masculin___________ | |
| **État civil :** Célibataire____ Marié(e)___ Divorcé(e)___ Veuf(**ve**)___ Partenariat___ | | |
| **Niveau d'éducation :** Aucun_____ Primaire_____ Secondaire____ Supérieur_____ | | |
| **Profession :** | | |
| **Zone de résidence :** Ville______________________ Quartier______________________ | | |

**QUESTIONNAIRE**

1. **Données sociodémographiques**
2. **Antécédents médicaux**

**Répondez aux questions suivantes en cochant la réponse qui vous convient le mieux. Dans la question 4**

| 1. **Depuis combien de temps suivez-vous un traitement d'hémodialyse** ? |
| --- |
| 1. **De quelle maladie souffrez-vous** ?   Néphropathie diabétique Glomérulonéphrite Néphropathie polykystique Trouble rénal congénital Insuffisance rénale aiguë Inssufissance renale chronique  Hypertension Si d'autres, précisez_______________________________ |
| 1. **A quelle fréquence êtes-vous soumis à l'hémodialyse** ?   1/ mois 2/ mois 1/semaine 2/semaine 3/semaine 4/semaine |
| 1. **Êtes-vous atteint d’une autre maladie** ?   Diabète Hypertension VIH/SIDA |
| 1. **Si autre Précise_________________________________________** |
| 1. **Des infections fongiques vous ont-elles été diagnostiquées au cours des deux dernières semaines** ? Oui Non |
| 1. **Avez-vous pris un antifongique au cours de la semaine écoulée ?** Oui Non |
| 1. Si oui, lequel ? ____________________________________________ |
| 1. **A quelle fréquence prenez-vous les antifongiques** ? Tous les jours 3fois/semaine   Au moins 1fois Chaque semaine |
| 1. **Quel type d'eau buvez-vous à la maison** ?   Eau Minérale Eau de fourrage Cours d’eau Eau de puits Eau du robinet |
| 1. **Combien de litre buvez-vous** ? <1L/jour Au moins 1,5L/jour >1,5L/jour |
| 1. **Avez-vous récemment changé de cathéter d'hémodialyse ?** Oui Non |
| 1. **Avez-vous remarqué des fuites avec votre cathéter** ? Oui Non |
| 1. **Avez-vous voyagé ce mois-ci ?** Oui Non   Si oui, où ? _________________________________________________ |

1. **Signes et symptômes**

**Répondez aux questions suivantes sur les signes et symptômes que vous avez pu ressentir en cochant oui ou non**

| **Questions** | **Oui** | **Non** |
| --- | --- | --- |
| 1. Avez-vous ressenti une douleur ou une gêne au niveau du bas-ventre ? |  |  |
| 1. Avez-vous eu une fièvre persistante au cours des 4 dernières semaines ? |  |  |
| 1. Avez-vous ressenti des démangeaisons, des rougeurs ou des irritations dans votre région génitale ? |  |  |
| 1. Avez-vous ressenti une sensation de brûlure en urinant ? |  |  |
| 1. Avez-vous remarqué un trouble inhabituel dans vos urines ? |  |  |
| 1. Vous êtes-vous senti fatigué ces deux dernières semaines ? |  |  |
